# Impact of a two‐stage rumen cannulation on the health and rumen function of six lactating dairy cows

**DOI:** 10.1111/vsu.14182

**Published:** 2024-10-30

**Authors:** Thomas Hartinger, Laura Beissel, Ezequias Castillo‐Lopez, Thomas Wittek, Johann Huber, Qendrim Zebeli

**Affiliations:** ^1^ Center for Animal Nutrition and Welfare University of Veterinary Medicine Vienna Vienna Austria; ^2^ Clinical Center for Ruminant and Camelid Medicine University of Veterinary Medicine Vienna Austria; ^3^ VetFarm Kremesberg University of Veterinary Medicine Pottenstein Austria

## Abstract

**Objective:**

To determine the impact of a two‐stage rumen cannulation on the health and rumen function of lactating dairy cows.

**Study design:**

Experimental study.

**Animals:**

Six lactating Holstein cows.

**Methods:**

We performed a two‐stage rumen cannulation in six Holstein cows that were 49 ± 11 days in milk. The following clinical health parameters and digestion‐associated variables were analyzed on seven measurement days, from before the first surgery until 28 days after the second surgery: body temperature, heart rate, respiratory rate, pain score, rumen fill score, fecal score, wet sieving, auscultation and palpation of the rumen, bodyweight, body condition score, and activity.

**Results:**

The pain score of the cows was constantly zero. Similarly, the body temperature and respiratory rate remained within physiological ranges, whereas the heart rate was slightly higher immediately after the second surgery. No differences were observed in rumen fill (2.00–2.67; *p* = .10) and fecal consistency scores (2.17–2.67; *p* = .42). The fecal particle size distribution showed negligible differences. The cows lost approximately 43 kg of bodyweight during the experiment (*p* < .01), which was reflected in a 0.5‐point body condition score loss (*p* < .01).

**Conclusion:**

A temporary minimal negative effect of a two‐stage rumenostomy on the health and body condition of early lactating Holstein cows was observed, whereas digestion was unaffected. Considering the limited sample size, further studies are required to substantiate these findings.

**Clinical significance:**

Given that animals are appropriately medically managed, experimental rumenostomy of lactating dairy cows may not compromise their health or rumen function.

## 1 INTRODUCTION

The unique symbiosis with a complex microbial community in the rumen enables ruminants to utilize fibrous feedstuffs, thereby enabling the production of valuable foods with highly bioavailable nutrients from non‐edible biomass. To improve our understanding of this sophisticated ecosystem and the digestion process in the rumen, research on the microbial community and its activity, as well as its interplay with the host, is necessary. Such research efforts have facilitated the optimization of cattle feeding in terms of productivity, health, and other aspects, such as greenhouse gas emissions.^1^

There are several options for obtaining rumen contents, including esophageal tubing, rumenocentesis, and permanent cannulation of the rumen. The first two methods may generally be suitable for determining variables such as rumen pH,^1^, ^2^, ^3^ whereas esophageal tubing may also serve as a tool for therapeutic transfaunation.^4^ However, both esophageal tubing and rumenocentesis can only provide snapshots of ruminal fluid, and frequent sampling over longer periods is difficult. Microbes and their metabolites vary substantially among different locations within the rumen,^2^, ^5^ indicating that such samples are not representative of the rumen ecosystem. Both methods can only provide free rumen liquid, whose composition clearly differ from that of particle‐associated rumen liquid and solid digesta.^5^

In contrast to esophageal tubing and rumenocentesis, rumen cannulation is a permanent method that allows the frequent collection of liquid and solid rumen contents in small and large volumes. It also facilitates sampling and investigation of rumen tissues without the need for slaughter. The positioning of feed in the rumen to study ruminal nutrient degradation, and therefore, to effectively optimize diets for ruminants, is only feasible in rumen‐cannulated animals.^6^ Despite these important utilities, rumen cannulation also constitutes an invasive intervention for animals^7^ and is repeatedly the subject of public controversy; it is designated as an indispensable part of research, but on the other hand is claimed as a cruelty to animals.^1^, ^8^

The process of rumen cannulation was described several decades ago (Philippson and Innes^9^), but so far, it has been largely applied as a means to an end in research. There is limited knowledge regarding whether this surgical intervention impairs the digestive function and health of ruminants. Schramm et al.^10^ recently described the uneventful postoperative recovery of male sheep after rumen cannulation without assessing the digestive function. Regarding large ruminants, only a summary of case reports on rumenotomy and rumenostomy as therapies for forestomach disorders in cattle is available,^11^, ^12^ and investigations on the impact of permanent rumenostomy on both health and rumen function in cattle are lacking. Such knowledge may be especially relevant for lactating dairy cows that are metabolically challenged, and insights gained from rumen cannulation in male sheep^10^ may not be fully applicable to lactating dairy cows. Therefore, this study accompanied the two‐stage rumen cannulation in six lactating Holstein cows to investigate whether and to what extent this surgical intervention affected clinical health and the variables associated with digestion. In this context, the physiological values of the health and digestion variables from scientific literature were applied as controls, that is, data obtained from healthy dairy cows that did not undergo any treatment.

## 2 MATERIALS AND METHODS

This study was approved by the Institutional Ethics and Animal Welfare Committee of the University of Veterinary Medicine Vienna and the national authority according to paragraph 26 of the Law for Animal Experiments, Tierversuchsgesetz 2012‐TVG (GZ: 2022–0.276.659).

### 2.1 Animals, housing, and feeding

The Holstein cows (3.41 ± 0.1 years of age) were on average 49 ± 11 days in milk of the second lactation when enrolled in the experiment. The cows were housed in a free‐stall barn equipped with 15 deep straw litter cubicles (2.6 × 1.25 m) at Teaching and Research Farm Kremesberg (VetFarm) in Pottenstein, Austria. They had continuous access to drinking water and were fed a silage‐based diet ad libitum (Table 1), which was prepared daily using an automatic feeding system (Trioliet Triomatic T15, Netherlands). Additionally, the cows received 3.0 kg of dairy concentrate (KuhKorn 19, Schaumann GmbH & Co. KG, Germany) in three meals per day. The cows were continuously monitored by trained staff, and no health issues requiring veterinary intervention occurred. The cows were milked twice daily in a double‐4 tandem milking parlor (DeLaval GmbH, Austria) at 6:00 a.m. and 5:00 p.m., and milk production was recorded daily using an electronic machine recorder (DeLaval Corp., Sweden).

**TABLE 1:** Composition of the diet fed to cows throughout the experiment.

Component	Proportion (%, dry matter basis)
Grass silage	38
Corn silage	33
Hay	5
Straw	2
Mineralized protein concentrate^a^	8
Mineralized grain mixture^b^	14

^a^ Rindastar 39 XP, Schaumann GmbH & Co KG, Germany.

^b^ Composition on a dry matter basis: 50% ground corn, 21% ground barley, 21% ground wheat, 3% limestone, and 5% Rindavit TMR 11 ASS‐Co + ATG (Schaumann GmbH & Co. KG, Germany).

### 2.2 Two‐stage rumen cannulation

The cows were subjected to a two‐stage rumen cannulation as described by Martineau et al.,^13^ with some modifications regarding the applied equipment and interim periods, as outlined below. Briefly, the animals underwent two surgeries. The rumen wall was sutured to the peritoneum and skin during the first surgery. Preoperatively, the animals' left flanks were clipped and subsequently wet‐shaved, and their access to feed was blocked the night before the first surgery. The cows were then administered a single intramuscular dose of amoxicillin (15 mg/kg; Vetrimoxin LA, Ceva Tiergesundheit GmbH, Germany) and ketoprofen (3 mg/kg; Rifen 10%, Vétoquinol GmbH, Austria). The surgical area was prepared in an area of approximately 30 × 50 cm on the left flank. Segmental anesthesia was induced using distal paravertebral local anesthesia and a reverse L‐block with procaine 2% (20 mL per injection site; Procamidor, Richter Pharma AG, Austria). The efficacy of anesthesia was verified by pricking the respective area with a needle approximately 10 min after the injection.

A circular line was marked on the skin using a petri dish with a 10 cm diameter to determine the site where the skin and muscle layers would later be removed. Guided by this line, the surgeon incised the skin to its full thickness and removed the skin flap from the subcutis, creating an opening in the skin. Using a similar approach, the muscles of the abdominal wall (external and internal oblique muscles and the transverse muscle) were incised and removed in a stepwise manner. All bleeding vessels were ligated. Finally, an incision was made through the fascia and peritoneum to open the abdominal cavity.

Subsequently, extraperitonealization of the rumen and its connection to the skin was performed. The rumen was then fixed using forceps. The peritoneum of the ruminal wall was sutured to the abdominal wall using a continuous suture pattern. The abdominal cavity was sealed by circularly suturing the rumen to the abdominal wall. The wound was cleaned daily as long as wound secretions required cleaning. A similar dosage of ketoprofen (3 mg/kg; Rifen 10%, Vétoquinol GmbH, Austria) was administered a second time 24 h after surgery.

Around 4 weeks later, the rumen wall tissue was excised following the circular skin and muscle wound after cleaning and local anesthesia (20 mL infiltrated into the rumen wall; Procamidor, Richter Pharma AG, Austria) during the second surgery. Before surgery, ketoprofen (3 mg/kg, Rifen 10%, Vétoquinol GmbH, Austria) was administered and antibiotics were not administered. After ligation of the bleeding blood vessels, a 4‐inch silicone rumen cannula with a rolled inner flange (BarDiamond Inc.) was inserted. All the surgeries were performed by the same surgeon.

### 2.3 Measurements

The cows were examined at seven different times one week before the first surgery (baseline), one and seven days after the first surgery, one day before the second surgery, one and seven days after the second surgery, and 28 days after the second surgery.

At each time point, the following variables were determined by a trained personnel at the same time on each examination day. Body temperature was determined rectally using a thermometer (Microlife VT 1831, Microlife Corporation, China) and was measured three times per day, at 8:00 a.m., 12:00 p.m., and 5:00 p.m., to consider the diurnal variation in body temperature.^14^ The respiratory rate was measured visually and the heart rate was measured by auscultation using a 3 M Littmann Classic III stethoscope (3 M, Germany). The stethoscope was used in combination with a percussion hammer (Hauptner Herberholz GmbH & Co. KG, Germany) and wooden plessimeter (Hauptner Herberholz GmbH & Co. KG, Germany) to detect potential aberrations in the forestomach system, such as right or left displaced abomasum or insufficient stratification of the rumen content. Three different scorings were performed: first, the cattle pain score was determined using the procedure of Gleerup et al.^15^ that is based on several indicators, such as “attention towards the surroundings,” “head position,” “ears position,” “facial expressions,” “response to approach,” and “back position;” second, the rumen fill was scored using the scheme of Zaaijer and Noordhuizen^16^; third, fecal consistency was scored according to Skidmore.^17^

On‐farm wet sieving of fresh fecal samples was performed as described by Kljak et al.^18^ and Khorrami et al.^19^ Briefly, approximately 200 g of feces was collected per cow in the morning of each measurement day and soaked in 1 L of tap water for 15 min. Subsequently, the samples were stirred and transferred onto a sieve cascade consisting of three sieves of decreasing pore sizes: 2.0, 1.18, and 0.5 mm (F. Kurt Retsch GmbH & Co. KG, Germany). The samples were sieved under a constant flow of tap water until clear water passed through the sieve cascade. After visually controlling the sieves, the particles retained on the sieves were directly weighed, and the proportional distributions were calculated.

The bodyweight (BW) and body condition score (BCS) of the cows were obtained at the baseline and on the last measurement day, that is, 28 days after the second surgery. Therefore, both variables were determined directly after morning milking, and the BCS was assessed by a single trained person following the instructions of Wildman et al.,^20^ but reported to the quarter point. The activity of the cows was continuously recorded using indwelling rumen sensors (that included an accelerometer) (pH Plus Bolus SX‐1042A, smaXtec Animal Care GmbH, Austria), which were recently validated for this purpose.^21^

### 2.4 Statistical analysis

The dataset was analyzed in SAS 9.4 (SAS Institute Inc.) using the Shapiro–Wilk normality method of PROC UNIVARIATE to validate the normal distribution. When the data for a specific variable were not normally distributed, they were logarithmically transformed or, if needed, in a second step square root transformed. All data were analyzed using PROC MIXED, with the measurement day as a fixed effect and the cow as a random effect. Measurements obtained from the same cow at different time points were considered repeated measurements. Therefore, an autoregressive (1) covariance structure was chosen. For body temperature, the fixed effect of the time of day was also included, as was its interaction with the fixed effect of the measurement day. Multiple mean comparisons were performed using the post hoc Tukey–Kramer test. The significance level was set at *p* = .05. Additionally, a posteriori power analysis with the present key response variables was conducted according to Stroup^22^ and Kononoff and Hanford,^23^ which displayed an average statistical power of 0.71 with a type I error of 0.05.

## 3 RESULTS

### 3.1 Variables related to health

**FIGURE 1:**
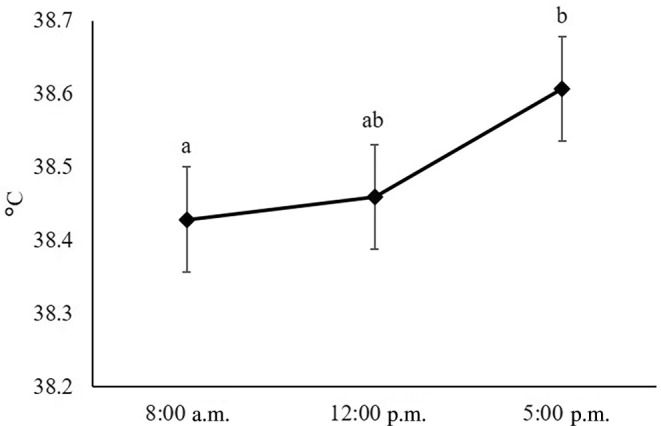
Diurnal variation in the body temperature in cows, determined rectally at 8:00 a.m., 12:00 p.m., and 5:00 p.m. Error bars indicate the standard error of the measurements and superscript letters denote differences between the least square means (*p* ≤ .05).

The body temperature ranged from 38.4–38.6°C between the measurement days that did not differ from each other (*p* = .40; Table 2), and there was no interaction between measurement day and time (*p* = .51). However, we observed a main effect of time on the body temperature (*p* = .04) with approximately 0.2°C higher values at 5:00 p.m. than at 8:00 a.m., (Figure 1).

**TABLE 2:** Digestion‐related variables in cows at different time points* during a two‐stage rumen cannulation.

	Surgery stage I			Surgery stage II						
	−7d	+1d	+7d	−1d	+1d	+7d	+28d	SEM	*p*‐Vvalue	Physiological range/value (reference)
Mean body temperature (°C)	38.5	38.4	38.5	38.5	38.6	38.5	38.5	0.08	0.40	38.3–38.8 (Baumgartner and Wittek^24^)
Pain score	0.00	0.00	0.00	0.00	0.00	0.00	0.00	‐	‐	0 (Gleerup et al.^15^)
Heart rate (/minute)	70.7.^b^	74.7^b^	71.3^b^	79.3^ab^	88.0^a^	71.3^b^	77.3^ab^	2.30	<0.01	60–80 (Baumgartner and Wittek^24^)
Respiratory rate (/minute)	26.3^b^	32.0^ab^	36.3^ab^	38.7^a^	34.0^ab^	37.7^a^	35.7^ab^	2.32	<0.01	10–30 (Baumgartner and Wittek^24^)
Activity†	10.41^a^	7.85^c^	9.65^abc^	11.0^a^	8.96^bc^	10.8^ab^	10.8^ab^	1.01	<0.01	10 (Lee et al.^21^)

*Note*: In each row, superscript letters indicate the difference between the least squares means (*p* ≤ 0.05).

* Time points were as follows: 1 week before the first surgery (−7 days; baseline), 1 day after the first/second surgery (+1 day), 7 days after the first/second surgery (+7 days), 1 day before the second surgery (−1 day), and 28 days after the second surgery (+28 days).

† Measured without specific unit from 0 to 100 (minimum–maximum).

As shown in Table 2, the pain score was consistently rated as 0.00 in all cows. The heart rate differed between the measurement days (*p* < .01) and was higher one day after the second surgery than seven days after the second surgery, baseline, and one and seven days after the first surgery. Similarly, the respiratory rate also differed between the measurement days (*p* < .01), with higher numbers one day before and seven days after the second surgery than at the baseline. The activity of cows was highest at the baseline and one day before the second surgery, and these two measurement days were also higher than one day after the first and second surgeries, both of which showed the lowest activity values (*p* < .01).

### 3.2 Variables related to digestion

The scores for rumen fill and fecal consistency were not different among the seven measurement days, ranging from 2.00 to 2.67 (*p* = .10) and 2.17 to 2.67 (*p* = .42), respectively (Table 3). The fecal wet sieving showed no differences for the fractions >3.15 mm (*p* = .08) and <0.5 mm (*p* = .30), whereas the fractions >1.18 mm (*p* < .01) and >0.5 mm (*p* = .03) were affected by the measurement day. Therefore, the fraction >1.18 mm was higher at the baseline and one day after the first surgery than one day before the second surgery, as well as seven and 28 days after the second surgery. The fraction >0.5 mm was higher one day after the first surgery than on the last measurement day, while the other measurement days did not differ. Palpation and percussion of the rumen on both sides of the body revealed no abnormalities.

**TABLE 3:** Digestion‐related variables in cows at different time points* during a two‐stage rumen cannulation.

	Surgery stage I			Surgery stage II						
	−7d	+1d	+7d	−1d	+1d	+7d	+28d	SEM	*p*‐Value	Physiological range/value (reference)
Rumen fill score	2.33	2.00	2.67	2.17	2.50	2.17	2.00	0.22	0.10	3 (Zaaijer and Noordhuizen^16^)
Fecal consistency score	2.33	2.50	2.67	2.33	2.67	2.67	2.17	0.21	0.42	3 (Skidmore^17^)
Fecal wet sieving (%)										
>3.15 mm	9.22	8.46	11.1	19.8	18.2	16.7	13.1	3.21	0.08	9.72 (Khorrami et al.^19^)
>1.18–3.15 mm	11.0^a^	12.8^a^	10.0^ab^	6.59^b^	9.41^ab^	7.96^b^	6.10^b^	1.04	<0.01	9.12 (Khorrami et al.^19^)
>0.5–1.18 mm	8.86^ab^	11.0^a^	9.94^ab^	7.64^ab^	7.73^ab^	8.45^ab^	7.41^b^	0.81	0.03	12.9 (Khorrami et al.^19^)
<0.5 mm	71.0	67.7	68.9	65.9	64.9	67.6	73.4	2.75	0.30	68.3 (Khorrami et al.^19^)

*Note*: In each row, superscript letters indicate the difference between the least squares means (*p* ≤ 0.05).

* Time points were as follows: 1 week before the first surgery (−7 days; baseline), 1 day after the first/second surgery (+1 day), 7 days after the first/second surgery (+7 days), 1 day before the second surgery (−1 day), and 28 days after the second surgery (+28 days).

### 3.3 Body condition and milk yield

**FIGURE 2:**
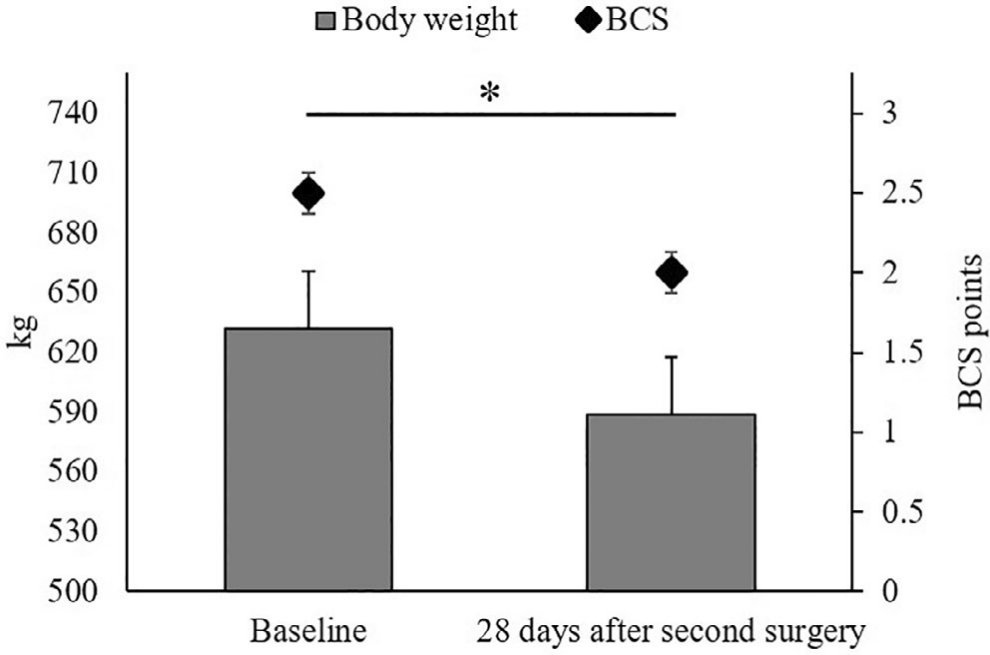
Changes in bodyweight and body condition score of cows measured at the baseline and 28 days after the second surgery. Error bars indicate the standard error of the measurements and the asterisk denotes a difference between the least square means (*p* ≤ .05).

**FIGURE 3:**
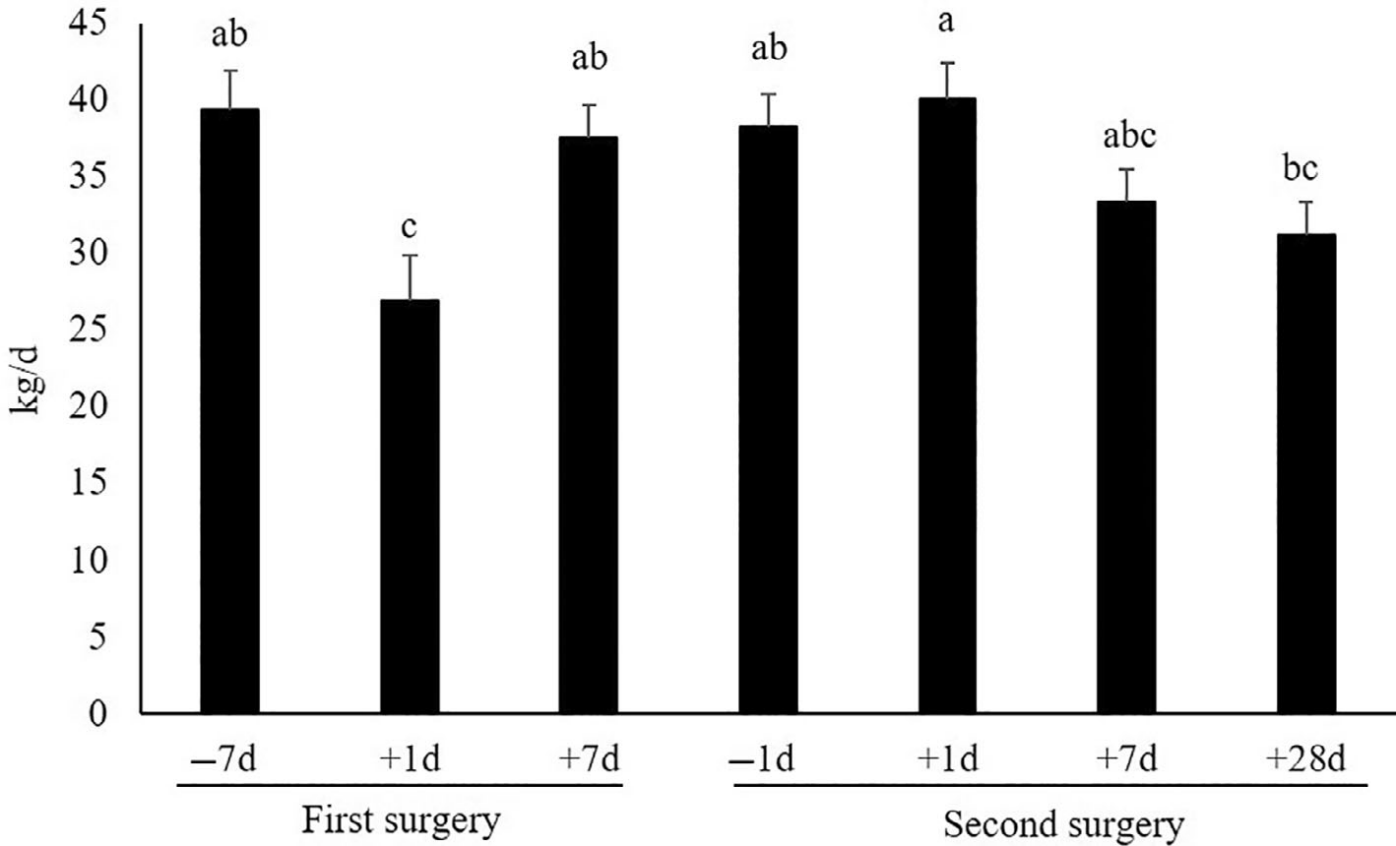
Daily milk yield of cows determined at different time points during a two‐stage rumen cannulation: one week before the first surgery (−7 days, baseline), one day after first/second surgery (+1 day), seven days after first/second surgery (+7 days), one day before the second surgery (−1 day), and 28 days after the second surgery (+28 days). Error bars indicate the standard error of the measurements and superscript letters denote differences between the least square means (*p* ≤ .05).

As illustrated in Figure 2, the BW of cows significantly decreased by approximately 43 kg from baseline measurement to 28 days after the second surgery (*p* < .01). Likewise, the BCS was 0.5 points lower at the end of the experiment when compared with the baseline (*p* < .01; Figure 2). Regarding milk yield (Figure 3), we observed differences among the time points (*p* = .01), with the highest milk yield of 40.0 kg/day one day after the second surgery and the lowest milk yield of 26.9 kg/day one day after the first surgery. Furthermore, milk yield was lower 28 days after the second surgery than seven days after the first surgery and one day before or after the second surgery.

## 4 DISCUSSION

This study aimed to evaluate the extent to which a two‐stage rumen cannulation method affects clinical health and digestion variables. Regarding clinical health, we found that the body temperature was in a physiological range of 38.3–38.8°C,^24^ suggesting no fever during the experiment. The diurnal variation in body temperature remained within this range, and higher values in the evening hours are a common observation in cattle,^14^ but are not related to rumen cannulation surgery. Future research efforts may also determine systemic inflammatory biomarkers, such as the acute phase proteins haptoglobin or serum amyloid A, to assess the state of subtle innate immune reactions.^25^ No signs of pain were detected using the pain evaluation approach described by Gleerup et al.^15^ that was specifically developed for lactating dairy cows and successfully demonstrated sufficient sensitivity and specificity, including detection of low‐grade pain in cattle. Despite being statistically different, the activity varied within the normal ranges reported in Holstein dairy cows that were not subjected to rumen cannulation or any other intervention.^21^ Therefore, these variables also provided no indication of compromised welfare or health, also not at a low‐grade pain level.

Notably, the heart rate increased to 88 beats/min one day after the second surgery, slightly above the physiological range of 60–80 beats/min,^24^ whereas the heart rate remained within the physiological range on all other measurement days. Hence, mild tachycardia one day after the second surgery may be a consequence of surgical intervention. Respiratory rates were assessed as physiological.^24^, ^26^ Nevertheless, we found certain variations in the respiratory rate, with higher numbers one day before and seven days after the second surgery than at the baseline. However, since the respiratory rate was also higher one day before the second surgery and the measurements around the first surgery did not differ from the baseline, it may be questioned whether these increments in respiratory rate can be truly related to surgical interventions. Indeed, one should consider that both surgeries were performed during the summer of 2022, and it is possible that the higher respiratory rates were caused by mild heat stress.^27^ The minimum and maximum air temperatures at 2 m height, obtained from the nearest weather station that is operated by the Austrian weather service (GeoSphere Austria, Vienna, Austria), were 12.3 ± 3.52°C and 24.2 ± 4.14°C, respectively, and hence in the range of mild heat stress for cattle.^28^

The rumen fill score, which is a common on‐farm indicator of feed intake in the last 24 h, was not different at the baseline versus the other measurement days, but with scores 2.00–2.50 being slightly below the optimum of 2.50–3.00 for lactating dairy cows.^16^ A similar observation was made for fecal consistency, which did not differ among the measurement days, but with scores marginally below the optimum of 3.00 for lactating dairy cows.^17^ Because these conditions were already observed at the baseline (i.e., before the first surgery), we hypothesized that these decreased scores were due to dietary factors, most likely a high intake of easily fermentable carbohydrates, along with a comparably low provision of physically effective neutral detergent fiber during the experiment. Therefore, no signs of maldigestion due to rumen cannulation were indicated by either of the score. Although fecal wet sieving showed statistically significant differences among the measurement days in certain fractions, they can be viewed as biologically negligible, as the fecal proportions of the present samples were similar to those obtained during wet sieving of feces from Holstein cows fed 40% concentrate and not undergoing rumen cannulation.^19^

Regarding the body condition of the cows, we observed a reduction in both BW and BCS from the baseline to 28 days after the second surgery. One possible explanation for this decrease in body tissue mass could be attributed to progressive negative energy balance. Our cows were at 49 ± 11 days in milk at the start of the experiment, and a negative energy balance obviously leads to body reserve mobilization, commonly occurring until the peak of lactation.^29^ However, there was a temporary decline in milk yield from the baseline to one day after the first surgery, which can be explained by the more intense character of the first surgery, during which the skin and muscle layers were removed,^13^ compared with the second surgery, when only the silicon rumen cannula was inserted. Another reason may be the removal of food the night before the first surgery. Since access to feed was not restricted before the second surgery, the absence of a milk yield decrease at this time point appears logical; thus, the potential impact of rumenostomy cannot be fully clarified. The fact that the milk yield of cows did not increase from the baseline to the end of the experiment, that is, when they should be approaching peak milk production, may actually be assessed as a negative response to rumenostomy. It can be speculated that the voluntary feed intake of the cows was not as high as it would have been without rumenostomy, likely because of some degree of discomfort following the surgical interventions, which then led to a constant milk yield, along with a reduction in BW and BCS. Future studies should investigate whether longer postoperative analgesia duration changes this pattern.

Once cannulated, ruminants typically have a much longer life expectancy than conspecifics kept in commercial livestock production systems,^6^ demonstrating that the presence of a rumen cannula is not a general threat to the animal.^7^ Considering the present data in its entirety, it became clear that the immediate rumenostomy process did not pose a general health risk, as suggested by Martineau et al.,^13^ and it did not compromise the digestion of lactating dairy cows. Therefore, although rumen cannulation is an invasive intervention, it does not appear to be in direct conflict with animal welfare or moral principles, especially when compared with other methods for obtaining ruminal fluid, such as esophageal tubing and rumenocentesis. From an animal welfare perspective, esophageal tubing and rumenocentesis result in significant stress in animals.^3^ Notably, this stress seems to be derived from animal handling and not from pain caused by the treatment itself, as local anesthesia does not change dairy cows' stress responses to rumenocentesis.^3^ In fact, both methods constitute exceptional interventions for which cows are naïve and cannot be trained, because they would likely result in health complications if performed frequently. In contrast, rumen‐cannulated animals can be handled and sampled with minimal stress and a low degree of restraint, which are important welfare aspects to consider when obtaining rumen samples.

In summary, the present data on clinical health parameters and variables associated with digestion that were recorded during a two‐stage rumen cannulation of six lactating Holstein cows provided little indication of a detrimental impact of the surgical intervention, that is, elevated heart rate one day after the second surgery. The milk production performance of cows reduced directly after the first surgery, and the decrease in body condition during the experiment may be related to the mobilization of body reserves during early lactation, potentially because of a generally lower feed intake. Therefore, given appropriate medical management, experimental rumenostomy appears ethically justifiable for research purposes in early lactation dairy cows, which has only been shown in small male ruminants.^10^ In the present study, all cows had the same genetic background and lactation status, experienced the same environmental conditions and treatments, and were continuously handled by the same personnel. Altogether, this should have resulted in very similar controlled conditions that reduced the variation in the dataset. This was also indicated by the satisfactory statistical power of 0.71 obtained from the posteriori power analysis. Nevertheless, considering the limited sample size of six animals, additional studies are required to substantiate our findings.

## AUTHOR CONTRIBUTIONS

Hartinger T, Dr agr, MSc, BSc: Conceptualization, data curation, formal analysis, investigation, methodology, supervision, visualization, writing – original draft, and writing – review and editing. Beissel L, Mag med vet: Data curation, formal analysis, writing ‐ review and editing. Castillo‐Lopez E, PhD, MSc, BSc: Investigation, writing ‐ review and editing. Wittek T, Prof Dr med vet, Diplomate ECBHM: Investigation, Writing ‐ review and editing. Huber J, Dr med vet: Investigation, writing ‐ review and editing. Zebeli Q, Prof Dr sc agr: Conceptualization, methodology, project administration, writing ‐ review and editing.

## FUNDING INFORMATION

This study did not receive any specific grants from funding agencies in the public, commercial, or non‐profit sectors.

## CONFLICT OF INTEREST STATEMENT

None of the authors have any financial or personal relationships that could inappropriately influence or bias the content of this paper.
